# Mathematical Modeling of Monovalent Permselectivity of a Bilayer Ion-Exchange Membrane as a Function of Current Density

**DOI:** 10.3390/ijms23094711

**Published:** 2022-04-24

**Authors:** Andrey Gorobchenko, Semyon Mareev, Victor Nikonenko

**Affiliations:** Membrane Institute, Kuban State University, 149 Stavropolskaya St., 350040 Krasnodar, Russia; gorobchenkoandrey@mail.ru (A.G.); mareev-semyon@bk.ru (S.M.)

**Keywords:** electrodialysis, cation-exchange membrane, selective ion transport, mathematical modeling, kinetic ion transfer control, permselectivity–permeability trade-off

## Abstract

Modification of an ion-exchange membrane with a thin layer, the charge of which is opposite to the charge of the substrate membrane, has proven to be an effective approach to obtaining a composite membrane with permselectivity towards monovalent ions. However, the mechanism of permselectivity is not clear enough. We report a 1D model based on the Nernst–Planck–Poisson equation system. Unlike other similar models, we introduce activity coefficients, which change when passing from one layer of the membrane to another. This makes it possible to accurately take into account the fact that the substrate membranes usually selectively sorb multiply charged counterions. We show that the main cause for the change in the permselectivity coefficient, *P*_1/2_, with increasing current density, *j*, is the change in the membrane/solution layer, which controls the fluxes of the competing mono- and divalent ions. At low current densities, counterion fluxes are controlled by transfer through the substrate membrane, which causes selective divalent ion transfer. When the current increases, the kinetic control goes first to the modification layer (which leads to the predominant transfer of monovalent ions) and then, at currents close to the limiting current, to the depleted diffusion layer (which results in a complete loss of the permselectivity). Thus, the dependence *P*_1/2_ − *j* passes through a maximum. An analytical solution is obtained for approximate assessment of the maximum value of *P*_1/2_ and the corresponding fluxes of the competing ions. The maximum *P*_1/2_ values, plotted as a function of the Na^+^ ion current density at which this maximum is reached, gives the theoretical trade-off curve between the membrane permselectivity and permeability of the bilayer monovalent selective ion-exchange membrane under consideration.

## 1. Introduction

To date, membrane methods of purification, separation and concentration are the most environmentally friendly and economically promising. One of these methods is electrodialysis (ED), the long-term use of which on an industrial scale confirms its effectiveness and expediency [1,2,3].

Conventional electrodialysis desalination, which involves removing any ions and thereby reducing the total salinity of a solution [1], is in demand, e.g., for wastewater treatment, the production of deionized water [4,5] and water for the food industry [6]. However, there are also applications for electrodialysis in which the removal of certain kinds of ions is important. For example, NaCl and KCl are removed from whey in the dairy industry [7], while calcium and organic ions are valuable components and their removal is undesirable. In upgrading groundwater for irrigation use, monovalent Na^+^ and Cl^−^ ions are removed, and multivalent hardness cations and sulfate anions, which are necessary for optimal plant growth, are retained [8]. Selectrodialysis is a novel kind of electrodialysis which uses a specifically designed stack containing monovalent-ion permselective ion-exchange membranes (IEMs) [9]. This new method essentially allows improving phosphate separation and recovery from wastewater [10], makes more effective industrially processed brine treatment [11] and resolves many other issues in the environment and the chemical and food industries [11,12,13,14,15,16,17,18]. The growing applications of membranes, the permeability of which for monovalent ions is much higher than for multivalent ones, has caused increased interest in the development of such IEMs and in understanding the mechanism of their specific permselectivity [13,19,20,21,22]. An important issue in this context is the so-called trade-off between membrane permselectivity and membrane permeability [13,19], which consists of the experimentally established fact that a change in membrane structure or properties that leads to an increase in permselectivity also leads to a reduction in the flux of preferentially transported species. This “trade-off” is closely related to the problem of the dependence of fluxes of competing ions across a membrane on current density or applied voltage. The specific permselectivity coefficient characterizing the relative ability of the membrane to selectively transmit monovalent ions (species 1) compared to divalent ions (species 2) is defined through the ratio of their fluxes *J_i_* (in mol∙m^−2^s^−1^) or their partial current densities *j_i_* (in A∙m^−2^) [23]:

(1) $$P_{1/2}=\frac{J_{1}c_{2}^{0}}{J_{2}c_{1}^{0}}=\frac{j_{1}C_{2}^{0}}{j_{2}C_{1}^{0}}=\frac{T_{1}C_{2}^{0}}{T_{2}C_{1}^{0}}$$

where $c_{i}^{0}$ and $C_{i}^{0}=\left|z_{i}\right|c_{i}^{0}$ are the molar (mol∙m^−3^) and equivalent (eq∙m^−3^) ion concentrations in the bulk solution, respectively.

The problem of the dependence of $P_{1/2}$ on the electric current density, *j*, was vigorously discussed in the 1960s and 1970s in connection with the development of a technology for obtaining table salt from sea water by electrodialysis. A great contribution to the study of the $P_{1/2}$ − *j* dependence was made by the authors of refs. [24,25,26,27,28]. At present, this issue is once again relevant due to the rapid development of technologies for the production and application of IEMs that are selective to singly charged ions [18,19,29,30,31,32,33]. The general principle for the manufacture of such membranes is the formation of a thin active surface layer, which serves as a barrier to the transfer of ions that are counterions to the substrate membrane. This barrier creates only insignificant resistance for singly charged ions but is a serious obstacle for two- and especially for three-charged ions. The ideology of creating this barrier goes back to the works of Sata [34]. Such a barrier can be formed by applying a hydrophobic film [35], as well as by applying a single layer [17,22,30,36,37,38] having fixed ions with a charge opposite to that of the substrate membrane, or by several surface layers (layer-by-layer) [17,39,40,41] with alternating charge signs of fixed ions. The best results are obtained when applying several binary layers: the permselectivity coefficient increases with an increase in the number of such layers, *n*, but when *n* > 10, the increase in *P*_1/2_ is no longer observed [18,42].

For monopolar IEMs, there is a continuous loss of selectivity transfer of the preferentially transferred ion with increasing current density [27,28,36,43,44]. This effect is due to a change in the control of the counterion transfer kinetics that occurs with increasing current density. At low currents, the fluxes of competing counterions are controlled by the membrane: usually divalent ions preferentially pass through the membrane due to their greater sorption by the membrane phase [45]. As the current increases, the membrane still controls the counterion–coion permselectivity, but the kinetic control of the permselectivity between two competing counterions is increasingly shifted to the depleted diffusion layer, in which there is no selective transport of competing ions [28,46,47]. However, in the case of an IEM modified with a surface layer with a charge that is opposite to that of the substrate membrane, the *P*_1/2_ value firstly increases with *j*, then the curve passes through a maximum, after which a further increase in current density leads to a drop in specific permselectivity [22,36].

Roghmans et al. [36] investigated the possible reasons for the decrease in the specific permselectivity of a commercial CMX cation-exchange membrane modified with a thin anion-exchange layer by increasing current density when treating a ternary electrolyte solution. Based on the results of numerical simulations, they found that the presence of defects in the modification layer (domains not coated with selective material) cannot be the cause of the experimentally observed reduced permselectivity at high currents. The authors [36] suggested that structural changes in the modification layer, such as swelling, can cause a decrease in the fixed ions’ charge density in the layer, thereby reducing ion repulsion and permselectivity.

Golubenko et al. [22] studied the permselectivity of an anion-exchange membrane (AEM) modified with a thin cation-exchange layer. They agree with the hypothesis made by Roghmans et al. [36] and offered a possible specific mechanism for reducing the concentration of fixed groups: when water splitting begins, some of the fixed SO_3_^−^ ions in the cation-exchange modification layer can transform into the protonated uncharged form, which reduces the density of the fixed charge. Golubenko et al. [22] also proposed another mechanism that can take place in the case of the transfer of SO_4_^2−^ ions, which at a high concentration of H^+^ ions in the cation-exchange layer can transform into the singly charged form HSO_4_^−^.

In our study, we developed a new one-dimensional, stationary Nernst–Planck–Poisson model that disregards water splitting, and on the basis of which a different (compared to Refs. [22,36]) explanation of the extremal form of the $P_{1/2}$ − *j* dependence for a bilayer membrane is given. We show that, at low current densities, counterion fluxes are controlled by transfer through the substrate membrane, as in the case of monopolar single-layer membranes. As the current increases, the kinetic control passes first to the modification layer (providing the selective transfer of monovalent ions), and then to the depleted diffusion layer. The latter, as in the case of monopolar membranes, leads to a complete loss of permselectivity. It follows from the simulation that the $P_{1/2}$ − *j* dependence also passes through a maximum, even when there is neither a decrease in the concentration of fixed ions in the modification layer, nor any water splitting.

## 2. Theoretical Part

### 2.1. Mathematical Model

**Figure 1:**
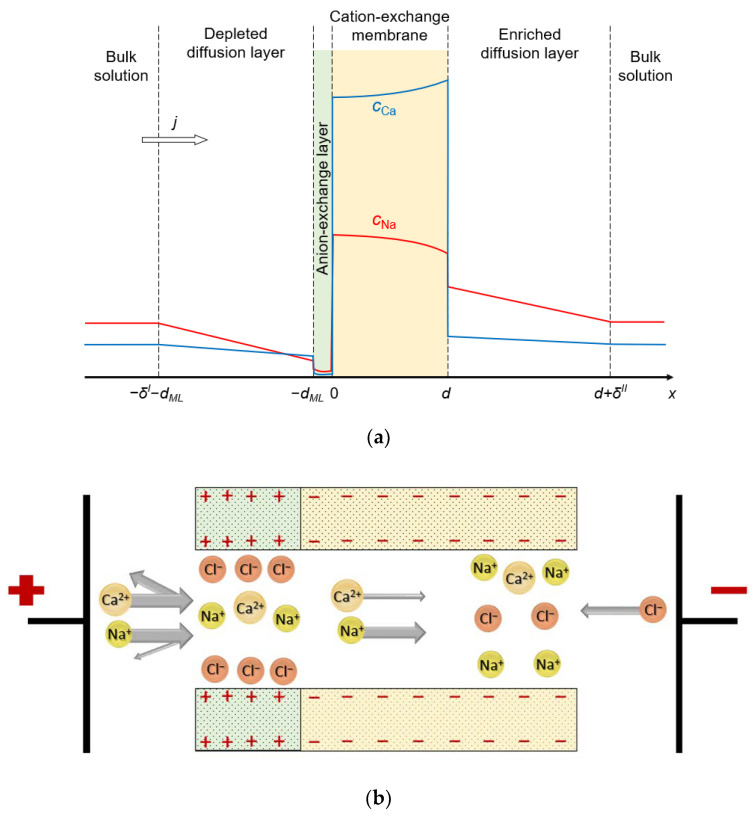
(**a**) Schematic representation of the simulated system, including the substrate CEM and anion-exchange modification layer of thicknesses *d* and *d_ML_*, respectively; depleted and enriched diffusion layers of thicknesses *δ^I^* and *δ^II^*, respectively, and the bulk solution. The colored lines show the concentration profiles of the monovalent counterions (red line) and divalent counterions (blue line). The direction of flow of electric current is shown by the arrow. (**b**) Diagram of a possible pore structure that corresponds to the simulated bilayer membrane.

The system under study consists of four layers: substrate CEM, thin anion-exchange modification layer, and two diffusion layers (DLs) adjacent to its surfaces. Two identical bulk solutions containing two kinds of cations (Na^+^ and Ca^2+^, for definitiveness) and one kind of anion (Cl^−^) flank the system under study (Figure 1).

The main assumptions in the formulation of this problem were similar to those in reference [44]:

- The substrate and modification layers are considered as a homogeneous medium in which fixed charged groups are uniformly distributed;
- It is assumed that the solvent flux through the membrane is negligible, and therefore the phenomena of osmosis and electroosmosis are not taken into account;
- The impact of convection transport in solution is taken into account implicitly through the diffusion layer thickness, which is considered independent of the voltage applied;
- The gradients of temperature, pressure and solution density are ignored;
- Water splitting and electroconvection are not taken into account.

The ion-exchange material of both layers can be represented as a charged sponge impregnated with a charged solution of the opposite sign. The membrane system can also be imagined as a bundle of nanometer pores with charged walls. One such pore is shown in Figure 1b: the wall charge on one edge of the pore is positive (anion-exchange modification layer) and on the other side it is negative (cation-exchange layer). Our model considers 1D ion transport along an axis normal to the membrane surface. It is possible to arrange such a distribution of the charged sites on the pore walls such that the average concentration of them in the cross section normal to the transport axis is the same as in the charged sponge representation. Thus, both images of the bilayer membrane, based on a charged sponge or a bundle of nanopores, can serve to visualize the 1D mathematical model developed in this paper.

The stationary transport of ions in the system under study is described by the Nernst–Planck–Poisson equations and the material balance equation:

(2) $$J_{i}^{}=-D_{i}^{}\left(\left(1+\frac{d\ln\gamma_{i}}{d\ln c_{i}}\right)\frac{dc_{i}^{}}{dx}-z_{i}c_{i}^{}E\right),$$

(3) $$\varepsilon \varepsilon_{0}\frac{dE}{dx}=F\left(\sum_{i=1}^{3}z_{i}c_{i}^{}+\bar{z}\bar{Q}\right)$$

(4) $$\frac{dJ_{i}}{dx}=0$$

here *J_i_*, *c_i_*, *D_i_*, *z_i_*, and *γ_i_* are the flux density, molar concentration, diffusion coefficient, charge number and activity coefficient of ion *i*, where *i* = 1 and *i* = 2 corresponds monovalent (Na^+^) и divalent (Ca^2+^) counterions, respectively, and *i* = 3 corresponds to Cl^−^ coion; *R* is the gas constant; *T* is the temperature; *F* is the Faraday constant; $E=-\frac{d\varphi }{dx}$ is the electric field strength; *φ* is the electric potential; *ε*_0_ is the vacuum permittivity; *ε* is the solution relative permittivity; $\bar{z}$ and $\bar{Q}$ are the charge number and concentration of fixed ion groups, respectively.

Equations (2)–(4) are valid for the DLs, substrate membrane, and modification layer. However, parameters $\bar{z}$ and $\bar{Q}$ depend on the coordinate: $\bar{z}$ and $\bar{Q}$ is set to zero in the DLs; $\bar{z}=z_{ML}$, $\bar{Q}=Q_{ML}$ in the modification layer; $\bar{z}=z_{m}$, $\bar{Q}=Q_{m}$ in the substrate membrane. The ion diffusion coefficients, *D_i_*, change in a similar way: $D_{i}=D_{i}^{s}$ in the DLs, $D_{i}=D_{i}^{ML}$ in the modification layer, $D_{i}=D_{i}^{m}$ in the substrate membrane. $D_{i}^{m}$ are found from the experimentally determined value of electrical conductivity; since the electrical conductivity of the modification layer cannot be measured, we set the $D_{i}^{ML}$ value *τ* times less than in solution, *τ* = 3 for all ions and cases considered. This parameter may be interpreted as a tortuosity factor. The $\gamma_{i}$ values are taken equal to unity in the DLs and modification layers, while in the substrate membrane $\gamma_{i}=\gamma_{i}^{m}$, where $\gamma_{i}^{m}$ can differ from 1. For a smoother change in these parameters at the boundaries of the respective layers, the weight boxcar function (rectangular wave) is used, while the thickness of all three interfacial transition regions is chosen to be 1 nm, which is close to the dense part of the electrical double layer [48]. A similar function was used by Evdochenko et al. [39] when modeling the charge density distribution of a polyelectrolyte multilayer membrane.

The current density, *j*, in the system includes the charges transported by all ionic fluxes:

(5) $$j=F\sum_{i}z_{i}J_{i}$$

The thicknesses of the depleted and enriched DLs are *δ^I^* and *δ^II^* respectively; all calculations were performed for *δ^I^* = *δ^II^* = *δ*. The origin of coordinates is set at the substrate membrane/modification layer interface (Figure 1a). It is assumed that the concentrations of the components in the bulk solution are known constants; the electric potential is set to zero at the left boundary of the system, and it is set to the value *φ*_0_ at the right boundary (potentiostatic electric mode):

(6) $$c_{i}\left(x=-\delta^{I}-d_{ML}\right)=c_{i}\left(x=d+\delta^{II}\right)=c_{i}^{0},$$

(7) $$\varphi \left(x=-\delta^{I}-d_{ML}\right)=0,$$

(8) $$\varphi \left(x=d+\delta^{II}\right)=\varphi_{0}$$

where *d* and *d_ML_* are the thicknesses of the membrane substrate and modification layer, respectively.

The functions *γ_i_c_i_*(*x*) and *φ*(*x*) are continuous throughout the four-layer system (between *x* = *−δ^I^ − d_ML_* and *x* = *d* + *δ^II^*), including the solution/modification layer interface and the modification layer/membrane interface.

Introduction to the consideration of the activity coefficients in Equation (2) and taking into account their continuous distribution in the layers of the system makes it possible to describe the selective sorption of individual kinds of ions by the membrane–substrate. This is the main difference between this model and those previously developed [22,36].

The system of Equations (2)–(4) with boundary conditions (6)–(8) is a boundary value problem for ordinary differential equations, the numerical solution of which was obtained using the commercially available software package COMSOL Multiphysics 5.6. The input parameters are listed in Table 1. The electric mode is set by the potential difference applied between the external edges of the left-hand and right-hand DLs (Figure 1a). The solution of the problem gives the value of the current density and partial current densities (fluxes) of both kinds of counterions and coions, as well as the distribution of the concentrations of all ions, the potential and the field strength. It is also possible to calculate the space charge density $\rho_{e}=F\left(\sum_{i=1}^{3}z_{i}c_{i}^{}+\bar{z}\bar{Q}\right)$ and the specific permselectivity coefficient, *P*_1/2_, Equation (1). The calculations are carried out for the case where the equivalent concentrations of both cations in the bulk solutions are the same and equal to 0.02 eq/L. The substrate membrane parameters are close to those of the homogeneous Neosepta CMX cation-exchange membrane studied in ref. [49]. The thicknesses of the depleted and the enriched DLs were selected based on the parameters of the experimental ED cell, which is typical in laboratory studies of electrodialysis ion separation [16].

### 2.2. Input Parameters

All the calculations were carried out using the input parameters presented in Table 1.

Comments and explanations on the methods applied to determine the input parameters are given in.

## 3. Results and Discussions

### 3.1. Limiting Current Density

The developed model describes the transfer of ions through a bilayer membrane bathed in a ternary electrolyte, two kinds of cations (Na^+^ and Ca^2+^) and one kind of anion (Cl^−^). The limiting fluxes of counterions, *J_i_*_lim_, are reached when the concentrations of all three kinds of ions become very small compared to their concentrations in the bulk solution. The fluxes of cations 1 and 2 can be determined by relation (9) [52], which is a generalization of the Peers equation [53]:

(9) $$J_{i\;\lim}=\frac{D_{i}^{s}c_{i}^{0}}{\delta^{I}}\left(1-\frac{z_{i}}{z_{3}}+\frac{z_{i}J_{3\;\lim}\delta }{z_{3}D_{3}^{s}c_{3}^{0}}\right),\;i=1,\;2,$$

where *J*_3 lim_ is the coion flux density through the membrane, which is directed opposite to the counterion fluxes (back electrodiffusion [54]), the value is taken at the limiting current density; note that *z*_3_ < 0. The term $\frac{z_{i}J_{3\;\lim}\delta }{z_{3}D_{3}^{s}c_{3}^{0}}$ reflects the fact that chloride ions, which leak back through the membrane and carry a negative charge, cause an additional transport of counterions from the depleted solution to the membrane surface (known as the exaltation effect [52,55]).

The summation of the fluxes expressed by Equation (9) (taking into account the charge of the ions) gives the limiting current density for the system under study, *j*_lim_:

(10) $$j_{\lim}=j_{\lim}^{0}+\frac{j_{3\;\lim}}{t_{3}^{0}},$$

where

(11) $$j_{\lim}^{0}=\frac{F}{\delta }\sum_{i=1}^{2}\left(\left(1-z_{i}/z_{3}\right)D_{i}^{s}z_{i}c_{i}^{0}\right)$$

is the limiting current density in the case of a membrane ideally impermeable to coions [46]; the second term on the right-hand side describes the effect of the exaltation of the limiting current of counterions, where *j*_3 lim_ is the limiting partial current density of the coion; $t_{3}^{0}=\frac{z_{3}^{2}D_{3}^{s}c_{3}^{0}}{\sum_{i=1}^{2}z_{i}^{2}D_{i}^{s}c_{i}^{0}}$ is a parameter resembling the coion transport number in solution.

### 3.2. Specific Permselectivity and CVC

**Figure 2:**
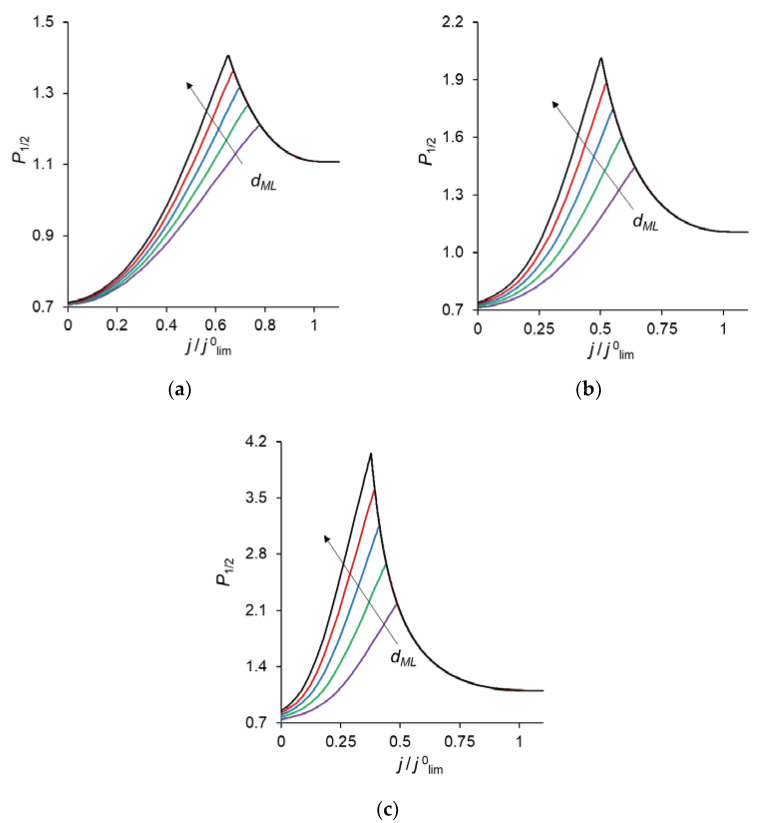
Dependencies of the specific permselectivity coefficient of the modified CEM on the electric current density with different *d_ML_* (10–30 nm) and *Q_ML_* = 0.5 M (**a**); 1 M (**b**); 2 M (**c**).

Figure 2 shows the specific permselectivity coefficient of a CEM modified with a thin anion-exchange layer as a function of the electric current density at different concentrations of fixed groups in the modification layer, *Q_ML_*, and its different thicknesses, *d_ML_*. It can be seen that at relatively low current densities, the *P*_1/2_ value increases, reaches a maximum value, and then gradually decreases at higher currents. Similar dependencies were obtained experimentally and theoretically by Golubenko et al. [22].

As can be seen from Figure 2, the value of *P*_1/2_ increases with increasing values of *d_ML_* and *Q_ML_*, as expected: the increase of both parameters leads to a higher resistance of the modification layer towards the divalent cations. In particular, an increase in *Q_ML_* results in the increasing Donnan exclusion of cations from the anion-exchange modification layer; since the Donnan exclusion is stronger for divalent coions, the increase in the barrier for the transfer of the divalent cations is essentially greater than for the monovalent cations.

**Figure 3:**
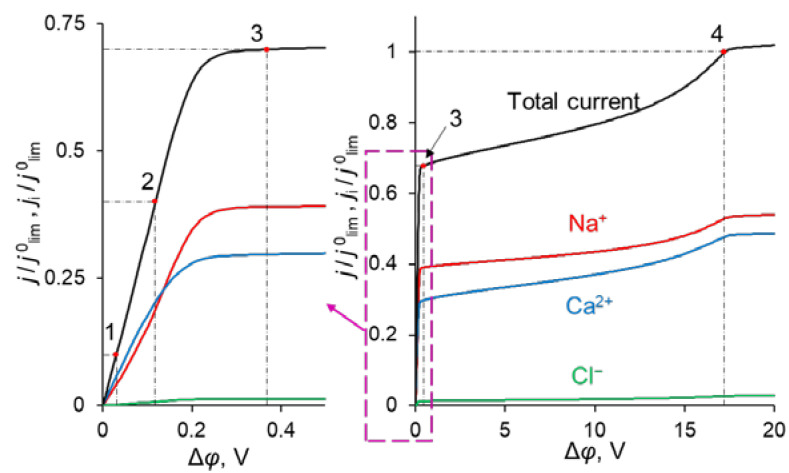
Theoretical CVC (black curve “Total current”) and partial CVCs (red «Na^+^», blue «Ca^2+^» and green «Cl^−^» curves) of the system with modified CEM at the parameters of the anion-exchange modification layer *Q_ML_* = 0.5 M and *d_ML_* = 20 nm. The numbers indicate the points on the CVC “Total current”, corresponding to *j* = 0.1 *j*^0^_lim_ (1); 0.4 *j*^0^_lim_ (2); 0.7 *j*^0^_lim_ (3); *j*^0^_lim_ (4).

The simulated total and partial current–voltage characteristics (CVCs) of the system under study are shown in Figure 3. It can be seen that at low voltages (up to 0.12 V corresponding to *j* = 0.4 *j*^0^_lim_, where *j*^0^_lim_ is defined in the previous section), the partial current of Na^+^ ion, *j*_1_, is lower than that of Ca^2+^, *j*_2_, that is *P*_1/2_ < 1. However, at *j* > 0.4 *j*^0^_lim_, *j*_1_ > *j*_2_, and moreover, the rate of growth of *j*_1_ is higher than *j*_2_ up to 0.36 V (*j* = 0.7 *j*^0^_lim_). The latter is expressed in increasing value of *P*_1/2_. At *j* > 0.7 *j*^0^_lim_, the situation changes: *j*_1_ increases at a lower rate than *j*_2_ and *P*_1/2_ decreases after passing through its maximum at *j* = 0.7 *j*^0^_lim_.

Point 1 on the total CVC corresponds to the typical current density (*j* = 0.1 *j*^0^_lim_), at which mass transfer is controlled by the substrate membrane. The total and partial CVCs in Figure 3 show two inclined plateau regions (the beginning of these plateaus is marked by points 3 and 4, respectively), where a great increase in voltage is needed to obtain a small increase in fluxes of both sodium and calcium ions. However, the increase in partial current of Na^+^ ions between points 3 and 4 is lower than that of Ca^2+^, which indicates a loss in *P*_1/2_ in this range of currents (Figure 2). Point 3 on the CVC corresponds to the electric current density (*j*/*j*^0^_lim_ ≈ 0.7), at which the maximum permselectivity is observed for the selected input parameters of the model (blue curve in Figure 2a). This point can also be related to the (first) limiting state, which is usually associated in electrochemistry (particularly, in electrodialysis) with the onset of a plateau on the CVC. As we can see in Section 3.3, this state corresponds to the depletion of ions at the modification layer/substrate interface. Point 4 corresponds to the depletion of ions at the solution/modification layer interface. It should also be noted that after the onset of the second limiting state (at *j*/*j*^0^_lim_ > 1), a further increase in current density (Figure 3) is due mainly to the transfer of coions through the substrate membrane and the related exaltation effect (Section 3.1). These effects cause only a slight decrease in *P*_1/2_ at *j*/*j*^0^_lim_ > 1 (Figure 2). Together, the coion transfer and the exaltation effect give a 5% increase in the total current value between Δ*φ* = 17 V (point 4) and Δ*φ* =30 V (not shown in Figure 3).

### 3.3. Concentration Profiles

**Figure 4:**
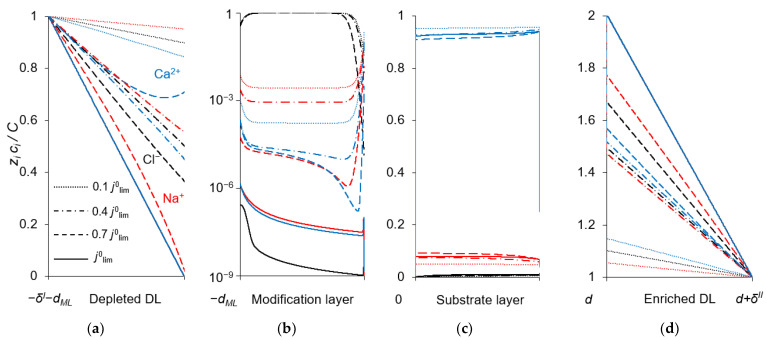
Concentration profiles of ions in the DLs (**a**,**d**), anion-exchange modification layer (**b**) and cation-exchange substrate membrane (**c**) at the parameters simulated for *Q_ML_* = 0.5 M, *d_ML_* = 20 nm and different electric current densities. *C* is the characteristic concentration in the considered layer: *C* = *c*^0^ (**a**,**d**), *C* = *Q_ML_* (**b**), *C* = *Q_m_* (**c**).

**Figure 5:**
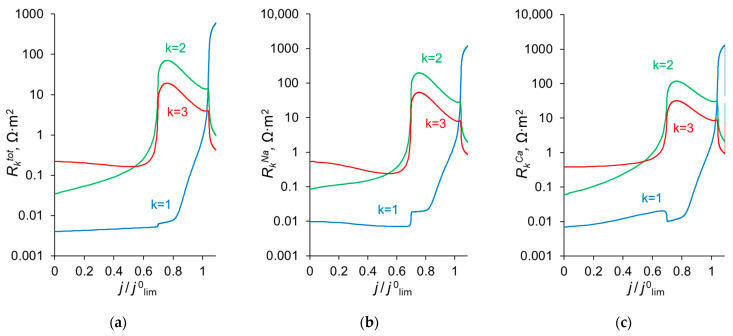
Differential resistance of layer *k* ($R_{k}^{tot}$) to the flow of the total current (**a**), as well as to the transfer of individual ions, Na^+^ ($R_{k}^{Na}$) and Ca^2+^ ($R_{k}^{Ca}$), (**b**) and (**c**), respectively. *k* = 1 relates to the depleted diffusion layer, *k* = 2, to the modification layer and *k* = 3, to the substrate membrane.

To explain the behavior of the dependence of the permselectivity coefficient on the current density (Figure 2), the concentration profiles for all three ions have been calculated at electric current densities corresponding to points (1–4) in Figure 3, in the case where *Q_ML_* = 0.5 M, *d_ML_* = 20 nm (Figure 4). For better comparison of the ion concentrations in the modification layer, the concentration profiles in it are presented on a logarithmic scale (Figure 4b). The animation of the changes in the ion concentration profiles in the depleted DL with an increase in the potential drop is presented in the Supplementary Materials (Animation S1). Figure 5 shows the dependencies of the differential resistance of individual layers (depleted DL, modification layer and substrate membrane) as a function of the ratio *j*/*j*^0^_lim_. Both the total resistance of the layer *k* (defined as $R_{k}^{tot}=\frac{d(\Delta \varphi_{tot})}{dj}$), and the resistance of the layer *k* with respect to Na^+^ and Ca^2+^ ions ($R_{k}^{Na}=\frac{d(\Delta \varphi_{tot})}{dj_{Na^{+}}}$ and $R_{k}^{Ca}=\frac{d(\Delta \varphi_{tot})}{dj_{Ca^{2+}}}$) are shown; *k* is the layer number: *k* =1 (depleted DL), 2 (modification layer), 3 (substrate layer).

Figure 4a shows that in the depleted DL, at current densities of 0.1 *j*^0^_lim_ and 0.4 *j*^0^_lim_, the concentration of Ca^2+^ ions at the surface of the modification layer decreases more than the concentration of Na^+^ ions, because the flux of Ca^2+^ ions exceeds the flux of Na^+^ ions (Figure 3). When passing from *j* = 0.4 *j*^0^_lim_ to *j* = 0.7 *j*^0^_lim_, the concentration of Na^+^ at the modification layer surface decreases even more, while that of Ca^2+^ ions increases (Figure 4a). This is due to the fact that the resistance of the modification layer, especially with respect to Ca^2+^ ions, increases sharply with increasing current density in the indicated range. However, at *j* > 0.7 *j*^0^_lim_ the resistance of the depleted DL begins to increase, and it increases to a greater extent with respect to Na^+^ (because the concentration of these ions has almost reached its minimum value near the modification layer surface) compared to Ca^2+^. As a result, in this range of current densities, a more significant increase in the calcium flux is observed compared to the sodium flux (Figure 3). With that, the *P*_1/2_ value decreases sharply and approaches its limiting value, determined by the following equation:

(12) $$P_{1/2}^{\lim}=\frac{D_{1}^{s}\left(1-\frac{z_{1}}{z_{3}}+\frac{z_{1}J_{3\;\lim}\delta }{z_{3}D_{3}^{s}c_{3}^{0}}\right)}{D_{2}^{s}\left(1-\frac{z_{2}}{z_{3}}+\frac{z_{2}J_{3\;\lim}\delta }{z_{3}D_{3}^{s}c_{3}^{0}}\right)}.$$

Equation (12) is obtained by substituting Equation (9), which give the fluxes of the competing ions at *j = j*^0^_lim_, into Equation (1), which defines the *P*_1/2_ quantity.

**Figure 6:**
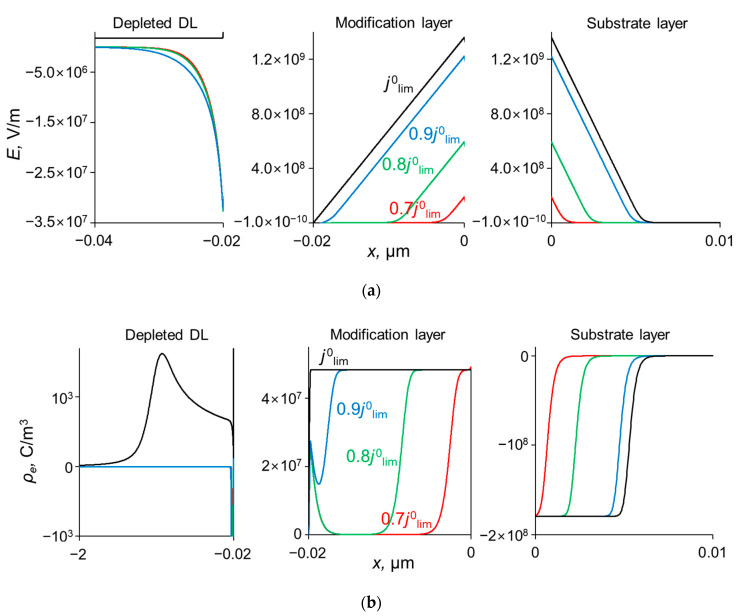
Profiles of the field strength, *E*, (**a**) and the space charge density, *ρ_e_*, (**b**) at the interfaces of the system under study at different current densities, *d_ML_* = 20 nm, *Q_ML_* = 0.5 M.

As Figure 3 shows, CVCs have two plateau regions that can be associated with the onset of two different limiting states. It follows from the comparison of Figure 3 and Figure 4, that the first limiting state (at *j* ≈ 0.7 *j*^0^_lim_ for the parameters presented in the legend to these figures) is reached when the concentration of Na^+^ and Ca^2+^ ions in the modification layer/substrate membrane interface (in the bipolar region) reaches critically low values (Figure 4b), causing a significant increase in the potential drop in the modification layer, in the membrane and in the membrane system as a whole when a small increase in current density occurs. A similar phenomenon is observed in systems with bipolar membranes: the limiting current occurs when the ion concentrations at the bipolar boundary approach zero [56,57]. At current densities *j* > 0.7 *j*_lim_, only a slight increase in the Na^+^ flux is observed when there is a significant increase in the flux of doubly charged Ca^2+^ ions (Figure 3). Therefore, the maximum value of *P*_1/2_ is reached at this state (*j* ≈ 0.7 *j*^0^_lim_); the *P*_1/2_ value decreases, when *j* > 0.7 *j*_lim_ (Figure 2a). The Ca^2+^ concentration in the electroneutral region of the modification layer is low at all current densities. This region in Figure 4b can be approximately identified as the domain where the reduced concentration of Cl^−^ ions is <1. At *j* = 0.7 *j*_lim_, its value at the right-hand boundary of this layer (always within the electroneutral region) becomes much lower than that at the left-hand boundary (Figure 4b). With increasing *j* above 0.7 *j*_lim_, the concentration of all ions in the bipolar region decreases, which causes an increase in the field strength (Figure 6a) and an expansion of the space charge region (Figure 6b). Note that in the modification layer, not only the concentrations of coions (Na^+^ and Ca^2+^), but also the concentration of counterion (Cl^−^) decreases nearly to zero (Figure 4b). When the concentrations of all ions approach zero, the ability of the modification layer to be selectively permeable for Na^+^ becomes significantly reduced. With a further increase in *j*, the concentration of both Na^+^ and Ca^2+^ ions in the solution at the surface of the modification layer sharply decreases, the resistance of the depleted DL grows and the control over cation fluxes gradually passes to this DL. As *j* approaches *j*_lim_, the second limiting state occurs when sufficiently low concentrations of all ions are reached at the depleted DL/modification layer interface (Figure 4a). At *j* = *j*_lim_, the *P*_1/2_ value is determined by the parameters of the diffusion layer and, only slightly, by the back electrodiffusion of Cl^−^ ions through the membrane, Equation (12).

The value of $P_{1/2}^{\lim}$, found from Equation (12) using the numerically found *J*_3lim_ at *j = j*^0^_lim_, is equal to 1.10, which is very close to the value of 1.11, found by direct numerical simulation (DNS) at Δ*φ* = 17 V. $P_{1/2}^{\lim}$ = 1.11 is reached in DNS at Δ*φ* = 40 V. Note that the *J*_3lim_ value has a small effect on $P_{1/2}^{\lim}$: the latter is equal to 1.12, if we put *J*_3lim_ = 0. It should also be noted that the values $P_{1/2}^{\lim}$ for the modified and unmodified membranes are equal. This qualitatively corresponds to the experimental results reported in references [30,37], as well as to the results of the numerical simulation in reference [22].

### 3.4. Analytical Assessement of the Maximum Value of P_1/2_

As our numerical simulation shows, there is a sharp maximum in the dependence of *P*_1/2_ on the current density. This maximum is achieved when the divalent cation concentration vanishes at the right-hand boundary of the modification layer adjacent to the substrate membrane. This state was identified above as the first limiting state of the membrane system. The flux of the monovalent cation in this state reaches a value $J_{1\max P}^{}$, which is close to its limiting value attained (in condition of *J*_3_ = 0) at *j* = *j*^0^_lim_, Equation (11). Therefore, taking into account Equation (9), we can write the following equation to evaluate the flux density of the monovalent cations at the point of maximum *P*_1/2_ value:

(13) $$J_{1\max P}^{}\approx J_{1\;\lim}^{0}=\frac{D_{1}^{s}c_{1}^{0}}{\delta }\left(1+\left|z_{1}/z_{3}\right|\right).$$

To obtain an assessment of the flux of the divalent cation at this point, $J_{2\max P}^{}$, we use Equations (A6) and (A9), deduced in the Appendix A, for the fluxes of this ion in the depleted DL and in the modification layer, respectively, in conditions of the first limiting state. Both fluxes are presented as functions of the divalent cation concentration at the depleted DL/modification layer interface, $c_{2}^{s}$. When equating these fluxes, we obtain an equation, which contains only one unknown quantity $c_{2}^{s}$. In our estimates, we set $K_{D}^{z_{i}}$ = 1, since the activity coefficients of all ions in the DL and the modification layer are equal to 1. Solving this equation (which is a quadratic equation with respect to $c_{2}^{s}$) yields:

(14) $$c_{2}^{s}=\frac{-b+\sqrt{b^{2}+4abc_{2}^{0}}}{2a}$$

where $a=\frac{D_{2}^{ML}\left(1+\left|z_{2}/z_{3}\right|\right)}{Q_{ML}d_{ML}}$, $b=\frac{D_{2}^{s}\left(1+\left|z_{2}/z_{3}\right|\right)}{\delta }$.

Substituting the solution expressed by Equation (A6) gives

(15) $$J_{2\max P}^{}=\frac{D_{2}^{s}\left(1+\left|z_{2}/z_{3}\right|\right)\left(c_{2}^{0}-c_{2}^{s}\right)}{\delta }.$$

As it follows from Equation (15), to have a maximum possible flux of divalent cations, $c_{2}^{s}$ should be as large as possible, approaching $c_{2}^{0}$. Equation (14) shows that for this, the value of *a* should be as small as possible. In other words, to obtain a high value of *P*_1/2_, the modification layer should be thick with a high concentration of fixed charges; the diffusion coefficient of the divalent cation should be small.

Knowing the flux densities of ions 1 [Equation (13)] and 2 [Equation (15)], and applying the definition of *P*_1/2_ [Equation (1)], we can find the maximum value $P_{1/2}^{\max}$, using $c_{2}^{s}$ calculated from Equation (14):

(16) $$P_{1/2}^{\max}=\frac{D_{1}^{s}\left(1+\left|z_{1}/z_{3}\right|\right)}{D_{2}^{s}\left(1+\left|z_{2}/z_{3}\right|\right)\left(1-c_{2}^{s}/c_{2}^{0}\right)}.$$

**Figure 7:**
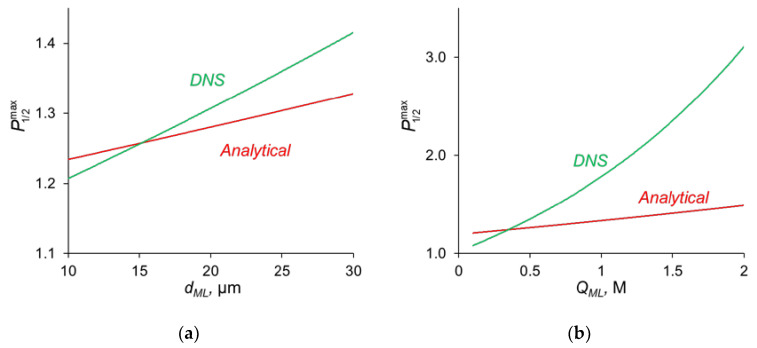
Dependence of $P_{1/2}^{\max}$, found by analytical calculations and by DNS on the thickness of the modification layer at *Q_ML_* = 0.5 M (**a**) and on the concentration of fixed ionic groups at *d_ML_* = 20 nm (**b**).

The comparison of the analytical calculations of $P_{1/2}^{\max}$, as described above, with the results of the direct numerical simulation (DNS) at different parameters of the modification layer are shown in Figure 7.

Figure 7a shows that with a change in the thickness of the modification layer, the analytical calculation has slight discrepancies with the results of the numerical calculation; however, the error does not exceed 10%, which is a very good result, taking into account all the assumptions made during the analytical calculation. A similar situation is observed when the concentration of fixed ionic groups of the modification layer is varied (Figure 7b) but only at *Q_ML_* ≤ 0.5 M. At high values of *Q_ML_*, there is a great deviation from the analytical calculation from the DNS. There are two main sources of this deviation. When deriving Equations (14)–(16), we assume, first, that the electrically neutral region occupies the main volume of the modification layer, and the ion concentrations at the outer edges of the charged interface are in local equilibrium. Second, a linear concentration distribution is assumed for the competing ions in the depleted DL. However, as Figure 4a shows, the latter is not fulfilled for the divalent cation (Ca^2+^) at *j* = 0.7 *j*^0^_lim_. The first assumption does not hold when the space charge interfacial regions are relatively large, which occurs at high *Q_ML_* values. See also the discussion in Section 3.7.

### 3.5. Change in the Kinetic Control with Increasing Current Density

**Figure 8:**
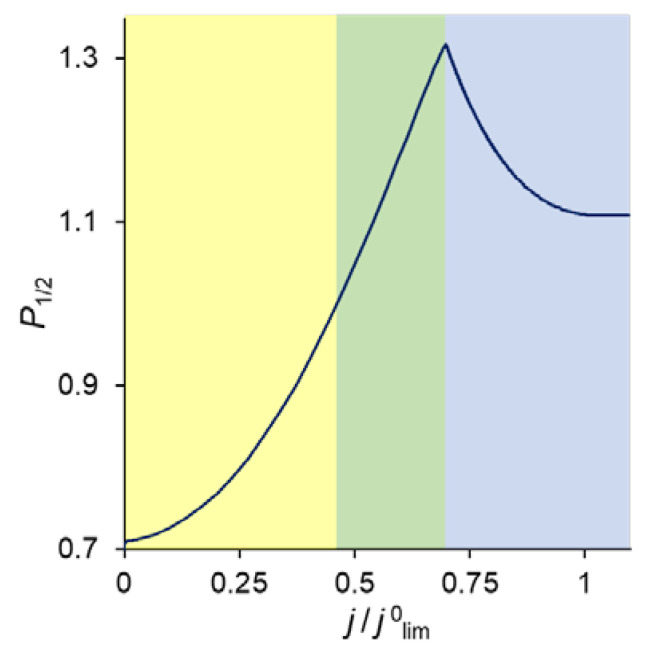
Specific permselectivity coefficient of a bilayer ion-exchange membrane bathed in a ternary electrolyte solution versus the ratio of the current density to its limiting value; simulation for *Q_ML_* = 0.5 M and *d_ML_* = 20 nm. The color of the shaded areas shows the electric current range, in which one of the layers controls the transfer of the competing cations: yellow refers to the substrate membrane control; green, to modification layer control; and blue, to depleted DL control.

As follows from the foregoing, the value of the permselectivity coefficient depends on which layer of the membrane system controls the counterion fluxes. Figure 8 illustrates this relationship. At low current densities, it is the substrate membrane which controls the fluxes since its resistance is the highest (Figure 5). In this current range, the divalent cation is preferably transferred through the bilayer membrane and *P*_1/2_ < 1. When the monovalent cation concentration at the modification layer becomes close to zero, and the Donnan exclusion of the divalent cations from this layer is strong enough, the resistance of this layer increases and the kinetic control passes to it. *P*_1/2_ becomes greater than 1, and rapidly increases with increasing current density. However, with that, the depletion of ions in the DL growth and its resistance increases correspondingly. The kinetic control passes to this depleted solution layer, which cannot deliver any selectivity to the transport of one of the competing ions. Consequently, the permselectivity is lost when the voltage is sufficiently great.

### 3.6. Trade-Off Curve between Membrane Permselectivity and Permeability. The Effect of the Concentration of Fixed Groups in the Modification Layer and Its Thickness

Figure 2 shows that with an increase in the thickness of the anion-exchange modification layer, *d_ML_*, the maximum value of *P*_1/2_ of the modified CEM increases; however, this maximum is observed at lower current densities. This dependence is explained by the fact that the presence of a layer on the membrane surface creates a barrier for the transfer of both counterions, but this barrier is higher for polyvalent ions due to their greater charge (the Donnan exclusion effect).

**Figure 9:**
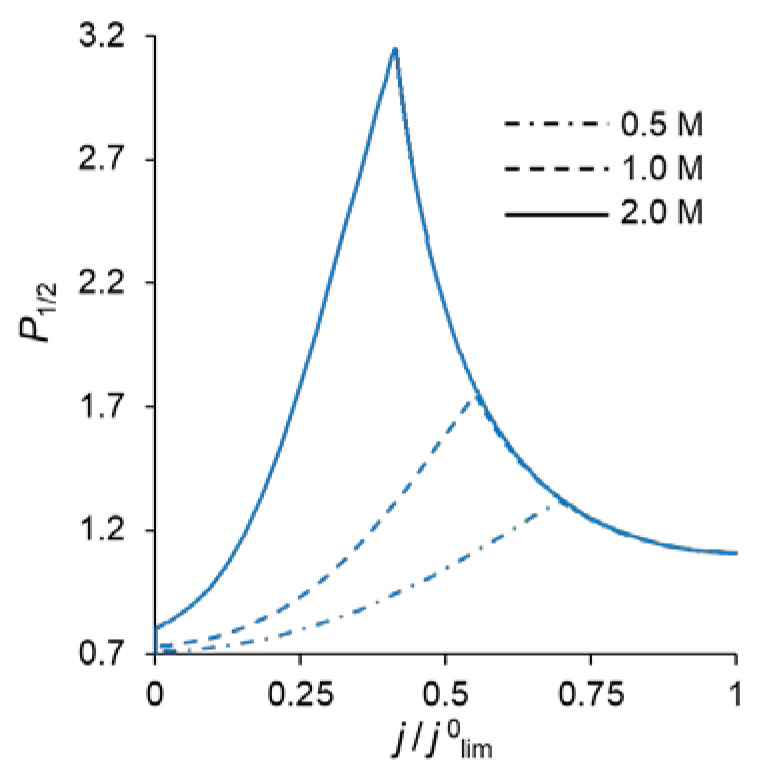
Dependencies of the permselectivity coefficient of the bilayer membrane on the electric current density at a fixed value of *d_ML_* (20 nm) and different *Q_ML_.*

Similarly, the value of *P*_1/2_ is affected by an increase in the concentration of fixed groups in the modification layer, *Q_ML_*. Figure 9 allows comparing the *P*_1/2_ − *j/j*^0^_lim_ dependencies at different values of *Q_ML_*. An increase in *Q_ML_* (and, as a consequence, the charge density of the fixed ions) leads to an increase in the forces of electrostatic expulsion of cations from the modification layer: the higher the charge number of these ions is, the more significant is the force of expulsion. That is why, with an increase in *Q_ML_*, the permselectivity of the modified membrane with respect to singly charged ions increases.

**Figure 10:**
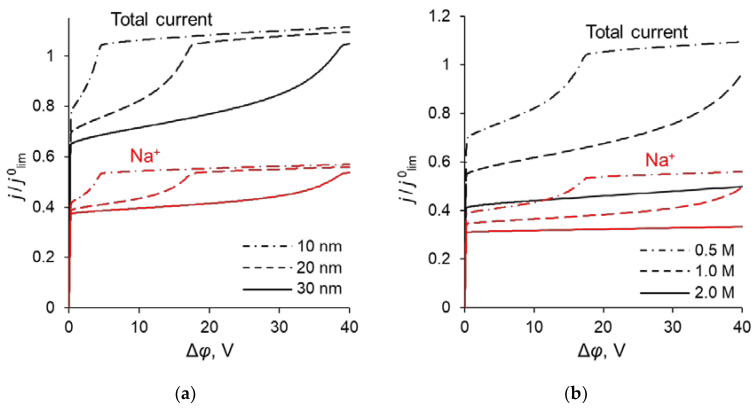
Total CVC of the system and partial CVCs of Na^+^ at different parameters of the modification layer: at different *d_ML_* (*Q_ML_* = 0.5 M) (**a**) and at different *Q_ML_* (*d_ML_* = 20 nm) (**b**).

**Figure 11:**
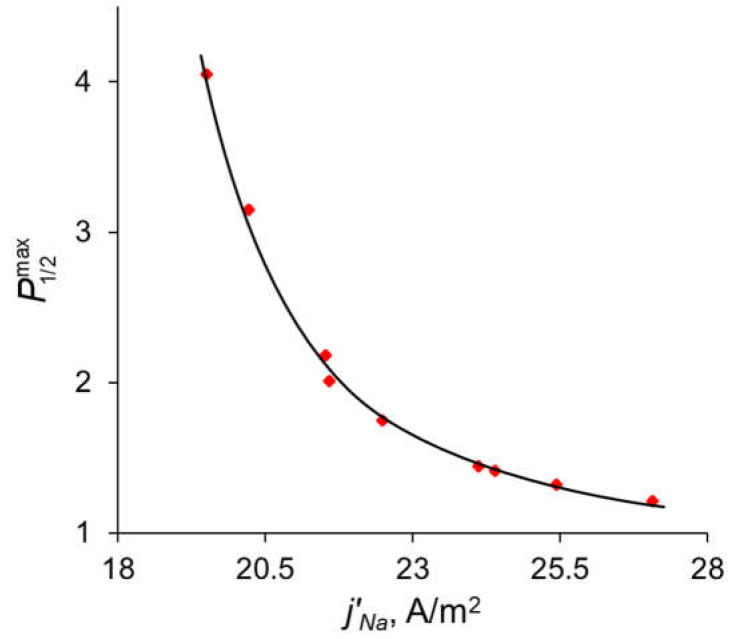
Dependence of the maximum value of the specific permselectivity coefficient on the partial current density of Na^+^. Red dots are the results of simulation at different values of *d_ML_* and *Q_ML_* (Table S1); the black line is the fitting curve for these results.

In the previous sections, it was shown that the maximum value of *P_1/2_* ($P_{1/2}^{\max}$) is reached when the sodium ion flux reaches its near-limiting value: when the current density/potential drop increases further, the flux of Na^+^ ions increases slightly compared to the flux of Ca^2+^. The dependency of the sodium ion partial current density along with the total current density as functions of the applied voltage are presented in Figure 10. The values of the partial current densities of Na^+^ and Ca^2+^ ions (*j’_Na_* and *j’_Ca_* respectively) corresponding to the maximum value of the specific permselectivity coefficient, $P_{1/2}^{\max}$, at different values of *d_ML_* and *Q_ML_* are presented in Table S1 (see Supplementary Materials). These results are shown also in Figure 11. Table S1 and Figure 11 testify that an increase in the thickness and concentration of the fixed groups in the modifying layer leads to an increase in the membrane permselectivity but also leads to a decrease in the flux of the preferably transferred ion. The dependence of $P_{1/2}^{\max}$ on the *j’_Na_*, calculated at different values of *d_ML_* and *Q_ML_*, is the so-called trade-off curve between membrane permselectivity and permeability for the case of monovalent-ion selective bilayer membranes. This kind of curve was first proposed by Robeson [58] as an empirical “upper bound” for gas-separation membranes. Later on this “upper bound” curve was repeatedly used to analyze the transport characteristics of many other types of membranes [13,18,19,22]. A short formulation of this trade-off relationship was formulated by Park et al. [19]: “highly permeable membranes lack selectivity and vice versa”.

### 3.7. Discussion

The application of the quasi-electro-neutral version of the model allows obtaining an analytical assessment of *P*_1/2_^max^, as mentioned above. However, at high values of the modification layer thickness and the concentration of fixed charges in this layer, the analytical assessment of *P*_1/2_^max^ deviates from the values found in DNS. One of the reasons for the deviation is the use of the local electroneutral assumption. In particular, this assumption stipulates that the ion concentrations at the outer edges of the charged interfaces between the modification layer and both neighboring layers are in local equilibrium. However, this equilibrium is not held when the thickness of these interfaces (which are interfacial electrical double layers) is relatively high. When deriving the equilibrium condition, it is usually assumed that the diffusion and electromigration components of an ion flux through the interface are both large in absolute value and opposite in sign. Then the difference between these components in the Nernst–Planck equation can be neglected, which leads to the Boltzmann equation and constancy of electrochemical potential for a given ion. The latter leads to the Donnan relations [59]. However, in the considered system the situation is rather special: the cations are coions for the modification layer, hence their fluxes usually should be low. However, it is not the case, and the cation fluxes are high; Figure 4a,b shows that the diffusion and electromigration components of the Ca^2+^ flux have the same direction in the left-hand SCR of the modification layer. Note also that a linear concentration distribution was assumed for the competing ions in the depleted DL. This assumption is not valid for the Ca^2+^ ion as well: due to the high resistance of the modification layer regarding this ion, its concentration increases when approaching the surface of the modification layer. Evidently, the latter effect of the concentration profile non-linearity increases with increasing the diffusion resistance of the modification layer towards cations, that is, with decreasing the value of the $a=\frac{D_{2}^{ML}\left(1+\left|z_{2}/z_{3}\right|\right)}{Q_{ML}d_{ML}}$ parameter entering Equation (14).

Therefore, we can see that the application of the local electroneutrality condition allows only an approximate description of the behavior of a bilayer membrane with respect to its permselectivity for monovalent ions. The adequacy of this version deteriorates with increasing modification layer resistance and the *P*_1/2_^max^ parameter values become significantly less than those given by DNS when using the Poisson equation (Figure 6). The Poisson equation makes it possible to take into account specific behavior of ions within the interfacial space charge regions at high current densities. However, deviations from adequacy are also possible when describing ion transport on a scale of less than 1 nm due to the fact that a model based on the NPP equations rests on the hypothesis of a dilute solution of point-like ions in a continuous medium. However, on such a small scale, ionic size becomes important (e.g., ionic crowding against a blocking surface), which must be taken into account when describing some phenomena such as the differential capacitance of the electrical double layer [60] and electroosmotic flow in (bio)microfluidic systems [61]. For this, modified electrokinetic equations for finite-sized ions can be used. Bazant et al. [60] gave a general framework of approaches involving such equations.

There is a certain similarity in the structure and behavior of artificial ion-exchange membranes and biological cell membranes. Both have pores/channels which are selectively permeable for some types of ions. Charge selectivity of cell membranes can be mediated by the electrostatic interaction of partially dehydrated permeating cations with negatively charged sites within a pore that is formed by side-chain carboxyl groups [62]. We study here an artificial composite bilayer membrane, one (thicker) layer of which bears fixed anions and another (thinner) layer has cations fixed to its matrix. One can imagine this membrane as a system of parallel long pores/channels. A short segment of such a pore would bear positive fixed charges on its walls, and its longer part would bear negative fixed charges on the wall. When two competing cations, such as Na^+^ and Ca^2+^, enter the short segment of the pore, the Ca^2+^ ions are repulsed by the positive charge of the walls, while Na^+^ can overcome this segment under the action of the gradient of concentration or electric potential. Furthering its way through the longer part of the pore with negatively charged walls would be rather easy for Na^+^. This long segment serves to stop the transfer of negatively charged anions, such as Cl^−^. The model presented in this paper describes monovalent-selective ion transport through such a composite pore/channel. We believe that this model can be useful not only for specialists in artificial membranes but also for those who study selective transport of ions through cell membranes. Mono- and divalent cation voltage–dependent permeation occurs in cyclic nucleotide-gated channels [63,64,65,66]; permselective mass transfer takes place in the plasma membranes of thermophilic bacteria [67] or in the aquaporin channels of cell membranes [68]. In particular, a simple expression for approximative evaluation of mono–divalent permselectivity can be useful for better understanding the causes and membrane parameters which control this selectivity.

## 4. Determination of the Model Input Parameters

### 4.1. Parameters of the Substrate Membrane and Modification Layer

The thickness, *d*, and the concentration of fixed groups, *Q_m_*, in the substrate membrane are taken the same as those for the commercial Neosepta CMX homogeneous cation-exchange membrane (produced by Astom, Tokuyama Soda, Japan) [50]. This membrane is preferably permeable to multivalent cations [49].

As mentioned above, the diffusion coefficients of ions in the substrate membrane, $D_{i}^{m}$, were calculated from the electrical conductivity and diffusion permeability of the Neosepta CMX membrane [50]. In the modification layer, the diffusion coefficients are taken 3 times fewer than in solution. This value was chosen from the consideration that usually the modification layer is not as dense as the substrate membrane, so the diffusion coefficients in it are only slightly less than those in solution [22,36].

### 4.2. Determination of Activity Coefficients

The relationship between the activity coefficients of counterions in solution and membrane follows from the condition of continuity of activities and electric potential at the solution/membrane interface, which leads to the ion-exchange equilibrium equation (also called the Nikolsky’s equation [69]):

(17) $$K_{21}={\left(\frac{c_{1}}{{\bar{c}}_{1}}\right)}^{1/\left|z_{1}\right|}{\left(\frac{{\bar{c}}_{2}}{c_{2}}\right)}^{1/\left|z_{2}\right|},$$

where ${\bar{c}}_{i}$ and $c_{i}$ are the molar concentrations of ions *i* in the membrane and solution, respectively.

The thermodynamic equilibrium constant *K*_21_ can be expressed in terms of the ion activity coefficients [45]:

(18) $$K_{21}={\left(\frac{\gamma_{1}^{m}}{\gamma_{1}}\right)}^{1/\left|z_{1}\right|}{\left(\frac{\gamma_{2}}{\gamma_{2}^{m}}\right)}^{1/\left|z_{2}\right|}.$$

However, when considering the laws of ion exchange for the membrane and solution phases, it is advisable to choose equivalent fractions of ions as concentration units, since only in this case are the activity coefficients of the components in the solution always equal to unity [45]. Then Equation (17) takes the form:

(19) $$\frac{{\bar{\theta }}_{2}{}^{1/\left|z_{2}\right|}}{{\bar{\theta }}_{1}{}^{1/\left|z_{1}\right|}}={\bar{K}}_{21}\frac{\theta_{2}{}^{1/\left|z_{2}\right|}}{\theta_{1}{}^{1/\left|z_{1}\right|}},$$

where ${\bar{\theta }}_{i}=\frac{\left|z_{i}\right|{\bar{c}}_{i}}{\left|z_{m}\right|Q_{m}}$ and $\theta_{i}=\frac{\left|z_{i}\right|c_{i}}{\left|z_{3}\right|c_{3}}$ are equivalent fractions of ion *i* in the membrane and solution, respectively; ${\bar{K}}_{21}=K_{21}{\left(\frac{\left|z_{m}\right|Q_{m}}{\left|z_{3}\right|c_{3}}\right)}^{1/\left|z_{1}\right|-1/\left|z_{2}\right|}$ is ion exchange equilibrium coefficient. For ion exchangers with sulfonate fixed groups ${\bar{K}}_{21}$ > 1, where subscript 1 refers to monovalent and subscript 2, to divalent cation [45].

A detailed derivation of Equations (17) and (19) is presented in the Supplementary Materials.

As was written earlier, the activity coefficients of the ions in the solution and in the modification layer are equal to unity; the activity coefficients of the counterions in the substrate membrane, $\gamma_{i}^{m}$, were selected so that the equivalent fraction of Ca^2+^ in the substrate membrane is approximately 20 times higher than Na^+^ (${\bar{K}}_{21}$ ≈ 20), when their equivalent concentrations in the equilibrium solution are the same and equal to 0.02 eq/L.

## 5. Conclusions

Within the framework of the developed 1D model based on the Nernst–Planck–Poisson equations, it was shown that the dependence of the specific permselectivity of the modified membrane on the electric current density passes through a maximum. The *P_1/2_ − j* dependence in this model is caused only by the transitions of the kinetic control of the transfer of competing ions from one layer of the multilayer membrane system to another. No changes in the concentration of fixed groups in the membrane–substrate and in the modification layer occur (these parameters are considered constant); accordingly, structural changes in the modification layer do not take place.

At low current densities, the transfer of cations through a CEM modified with a thin anion-exchange layer is controlled by the substrate membrane due to its relatively large thickness and resistance. Since this layer selectively absorbs doubly charged cations, these cations are selectively transported through the multilayer system. The increase in the specific permselectivity of the modified membrane with increasing current density is due to the transition of the kinetic control of mass transfer from the substrate membrane to the anion-exchange modification layer, which is a very significant barrier to calcium ion transfer and a less significant barrier to sodium ion transfer. This increase is observed until the system reaches the first limiting state due to a drop in the concentrations of Na^+^ and Ca^2+^ near the modifying layer/membrane–substrate interface (near the bipolar region) to critically low values by analogy with bipolar membranes. After reaching the first limiting state, the transfer of sodium ions almost does not increase, while a noticeable increase in the flow of calcium ions is observed. In this case, the concentrations of all ions (especially calcium ions) in the solution at the boundary with the modification layer rapidly decrease, and the kinetic control of mass transfer passes from the modification layer to the depleted diffusion layer. The second limiting state occurs when the concentrations of all ions at the depleted DL/modification layer interface reach critically low values. In this case, the value of *P_1/2_* reaches the same value that it reaches for the unmodified substrate membrane: the value of *P_1/2_* is determined by the parameters of the diffusion layer and depends very weakly on the reverse transfer of chloride anions from an enriched solution to a depleted solution.

An increase in the thickness and concentration of the fixed groups of the modification layer, on the one hand, allows a significant reduction in the flux of multivalent ions and an increase in membrane monovalent permselectivity. However, this also leads to a decrease in the flux of monovalent ions, and thus to a reduction in membrane permeability. This relationship is expressed by the simulated trade-off curve between membrane permselectivity and permeability for the case of monovalent-ion selective bilayer membranes.

## Figures and Tables

**Table 1:** Input parameters used in the calculations.

Parameter	Value	Description	Reference
*C* ^0^	0.02 eq/L	Bulk solution concentration	[16]
*Q_m_*	1.86 mol/L	Concentration of fixed ion groups in the substrate layer	[50]
*Q_ML_*	0.5 mol/L 1 mol/L 2 mol/L	Concentration of fixed ion groups in the thin modification layer	^*^
$D_{1}^{s}$	1.33·10^–9^ m^2^/s	Ion diffusion coefficients in the solution	[51]
$D_{2}^{s}$	7.96·10^−10^ m^2^/s		
$D_{3}^{s}$	2.04·10^−9^ m^2^/s		
$D_{1}^{m}$	6.57·10^−11^ m^2^/s	Ion diffusion coefficients in the substrate layer	[50]
$D_{2}^{m}$	2.50·10^−12^ m^2^/s		
$D_{3}^{m}$	2.89·10^−11^ m^2^/s		
τ	3	“Tortuosity factor” in modification layer	**
$\gamma_{1}^{m}$	1.5	Ion activity coefficients in the substrate layer	Equation (18)
$\gamma_{2}^{m}$	0.5		
$\gamma_{3}^{m}$	1		
*δ*	150 μm	Diffusion layer thickness	[16]
*d*	183 μm	Substrate layer thickness	[50]
$d_{ML}$	10–30 nm	Modification layer thickness	*
${\bar{K}}_{21}$	20	Ion-exchange equilibrium coefficient	[45]

* Variable parameters. ** Fitting parameters.

## Data Availability

Not applicable.

## Supplementary Materials

The following are available online at: https://www.mdpi.com/article/10.3390/ijms23094711/s1.

## Appendix A

Let us consider a ternary electrolyte composed of two kinds of cations, 1 and 2, and one kind of anion, 3.

The Nernst–Planck equations for the fluxes of these ions in a diffusion layer read:

(A1) $$J_{1}^{s}=-D_{1}^{s}\left(\frac{dc_{1}}{dx}+z_{1}c_{1}\frac{F}{RT}\frac{d\varphi }{dx}\right),$$

(A2) $$J_{2}^{s}=-D_{2}^{s}\left(\frac{dc_{2}}{dx}+z_{2}c_{2}\frac{F}{RT}\frac{d\varphi }{dx}\right),$$

(A3) $$J_{3}^{s}=-D_{3}^{s}\left(\frac{dc_{3}}{dx}+z_{3}c_{3}\frac{F}{RT}\frac{d\varphi }{dx}\right).$$

After dividing each of the above equations by $D_{i}^{s}$, summing the results, and taking into account the electroneutrality condition

(A4) $$z_{1}c_{1}+z_{2}c_{2}+z_{3}c_{3}=0,$$

we find:

(A5) $$\frac{J_{1}^{s}}{D_{1}^{s}}+\frac{J_{2}^{s}}{D_{2}^{s}}+\frac{J_{3}^{s}}{D_{3}^{s}}=-\frac{d\left(c_{1}+c_{2}+c_{3}\right)}{dx}=-\frac{\left(1+\left|z_{1}/z_{3}\right|\right)dc_{1}}{dx}-\frac{\left(1+\left|z_{2}/z_{3}\right|\right)dc_{2}}{dx}.$$

The last equality in Equation (A5) is obtained after eliminating *c*_3_ using Equation (A4). Since *J_i_* does not change along the coordinate *x* in the stationary state, Equation (A5) can be easily integrated over the thickness of the diffusion layer. Assume that the finite-difference approximation to concentration gradients can be applied in the diffusion layer, $dc_{i}/dx\approx \left(c_{i}^{0}-c_{i}^{s}\right)/\delta$, where $c_{i}^{s}$ is the concentration of ion *i* in the electroneutral region of solution at the membrane surface (at the surface of the modification layer in the case of bilayer membrane under study). Let *J*_3_ = 0, the membrane is impermeable to the anions. Since the concentrations of cations 1 and 2 can vary independently of each other, we can relate the first term on the left side of Equation (A5) to the first term on the right side of this equation; the same is true for the second terms. Then we obtain for cation *i*:

(A6) $$J_{i}^{s}=\frac{D_{i}^{s}\left(1+\left|z_{i}/z_{3}\right|\right)\left(c_{i}^{0}-c_{i}^{s}\right)}{\delta },\;i=1,\;2.$$

Applying the same assumptions for the modification layer of the bilayer membrane, we find:

(A7) $$J_{i}^{ML}=\frac{D_{i}^{ML}\left(1+\left|z_{i}/z_{3}\right|\right)\left({\bar{c}}_{i}^{s}-{\tilde{c}}_{i}^{s}\right)}{d_{ML}},$$

where ${\bar{c}}_{i}^{s}$ and ${\tilde{c}}_{i}^{s}$ are the molar concentration of ion *i* in the modification layer at the boundaries with the left-hand DL and the substrate membrane, respectively. At low $c_{i}^{s}$ concentrations, ${\bar{c}}_{i}^{s}$ can be expressed from the Donnan relation, as follows [70]:

(A8) $${\bar{c}}_{i}^{s}=\frac{{\left(c_{i}^{s}\right)}^{2}K_{D}^{z_{i}}}{Q_{ML}}.$$

As the numerical simulation shows, the *P*_1/2_ value reaches its maximum when the first limiting state is attained. In this state, the flux of Na^+^ is very close to its maximum possible value, and the Ca^2+^ concentration at the modification layer/substrate boundary is very small. Substituting Equation (A8) and condition ${\tilde{c}}_{i}^{s}$ = 0 into Equation (A7), we find:

(A9) $$J_{i}^{ML}=\frac{D_{i}^{ML}K_{D}^{z_{i}}\left(1+\left|z_{i}/z_{3}\right|\right){\left(c_{i}^{s}\right)}^{2}}{Q_{ML}d_{ML}}.$$
