# Supplementary material for: Mathematical Modeling of Monovalent Permselectivity of a Bilayer Ion-Exchange Membrane as a Function of Current Density
Source: Int J Mol Sci. 2022 Apr 24;23(9):4711. doi: 10.3390/ijms23094711 (PMC9104382; doi:10.3390/ijms23094711)
Supplement: Supplementary file 1 [file ijms-23-04711-s001.zip › Table S1.pdf]

Supplementary materials to the manuscript A. Gorobchenko et al.

Mathematical Modeling of Monovalent Permselectivity of a Bilayer Ion-Exchange Membrane as a Function of Current Density

S1. Evolution of the ion concentration profiles in the depleted diffusion layer with increasing potential drop

Animation of changes in the ion concentration profiles in the depleted diffusion layer with an increase in the potential drop is presented in the separate attached file (Animation S1).

S2. Values of j'_{Na}, j'_{Ca} and $P_{Na/Ca}^{\max}$ for various parameters of the anion-exchange modification layer

The values of the partial current densities of Na^+ and Ca^{2+} (j'_{Na} and j'_{Ca} respectively) corresponding to the maximum value of the specific permselectivity coefficient, $P_{Na/Ca}^{\max}$, as well as the themselves values of $P_{Na/Ca}^{\max}$ for various parameters of the modification layer (thickness, d_{ML} and concentration of fixed ionic groups, Q_{ML}) are presented in Table S1.

Table S1. Values of j'_{Na}, j'_{Ca} and $P_{Na/Ca}^{\max}$ for various parameters of the anion-exchange modification layer

Q_{ML}, M	d_{ML}, nm	j'_{Na}, A/m^2	j'_{Ca}, A/m^2	$P_{Na/Ca}^{\max}$
0.5	10	27.06	22.36	1.21
	20	25.43	19.56	1.32
	30	24.39	17.28	1.41
1.0	10	24.12	16.69	1.44
	20	22.49	12.84	1.75
	30	21.58	10.69	2.01
2.0	10	21.52	9.715	2.18
	20	20.21	6.390	3.15
	30	19.50	4.760	4.05

S3. Nikolsky's equation. Connection of the thermodynamic equilibrium constant with the coefficient of ion-exchange equilibrium

The distribution of ions between two phases (solution and membrane phases) that are in equilibrium depends on the difference in the electrochemical potentials of both phases. At equilibrium, the electrochemical potentials, μ_i, of ions of kind i in the membrane-solution phases are equal [S1]:

$$\bar{\mu}_i = \mu_i \ . \quad \text{(S1)}$$

From the definition of the electrochemical potential follows:

$$RT \ln a_i + z_i F\varphi = RT \ln \bar{a}_i + z_i F\bar{\varphi} \ , \quad \text{(S2)}$$

where $a_i = c_i \gamma_i$ and $\bar{a}_i = \bar{c}_i \bar{\gamma}_i$ are activity of ion i in the solution and in the membrane, respectively; c_i and γ_i are the molar concentration and activity coefficient of ion i in the solution, respectively; $\bar{c}_i$ and $\bar{\gamma}_i$ are the molar concentration and activity coefficient of ion i in the membrane, respectively; z_i is the charge number of the ion i, φ и $\bar{\varphi}$ are the electric potential in the solution and in the membrane, respectively; R is the gas constant; T is the temperature; F is the Faraday constant.

Expressing from equation (S2) the electrical potential drop between two phases, we obtain:

$$\bar{\varphi} - \varphi = -\frac{RT}{z_i F} \ln \frac{\bar{c}_i \bar{\gamma}_i}{c_i \gamma_i} \ . \quad \text{(S3)}$$

Let us apply equation (S3) to the system with the ternary electrolyte, where i = 1, 2 are counterions of various kinds (for example, singly and doubly charged counterions):

$$\frac{RT}{|z_1|F}\ln\frac{\bar{c}_1\bar{\gamma}_1}{c_1\gamma_1}=\frac{RT}{|z_2|F}\ln\frac{\bar{c}_2\bar{\gamma}_2}{c_2\gamma_2}, \tag{S4}$$

$$\left(\frac{\bar{c}_1\bar{\gamma}_1}{c_1\gamma_1}\right)^{\frac{1}{|z_1|}}=\left(\frac{\bar{c}_2\bar{\gamma}_2}{c_2\gamma_2}\right)^{\frac{1}{|z_2|}}. \tag{S5}$$

From equation (S5) it is easy to express the Nikolsky equation:

$$\frac{\bar{c}_2^{\,1/|z_2|}}{\bar{c}_1^{\,1/|z_1|}}=K_{21}\frac{c_2^{\,1/|z_2|}}{c_1^{\,1/|z_1|}}, \tag{S6}$$

where K_{21} is the thermodynamic equilibrium constant, which can be expressed in terms of standard chemical potentials, μ_i^0, [S2] or, according to equations (S5) and (S6), in terms of ion activity coefficients [S1]:

$$K_{21}=e^{\frac{\left(\frac{\mu_2^0}{|z_2|}-\frac{\mu_1^0}{|z_1|}\right)-\left(\frac{\bar{\mu}_2^0}{|z_2|}-\frac{\bar{\mu}_1^0}{|z_1|}\right)}{RT}}=\left(\frac{\gamma_1^m}{\gamma_1}\right)^{\frac{1}{|z_1|}}\left(\frac{\gamma_2}{\gamma_2^m}\right)^{\frac{1}{|z_2|}}. \tag{S7}$$

When considering the laws of ion exchange for the phases of the membrane and the solution, it is most expedient to choose equivalent fractions as units of concentration, since only in this case the activity coefficients of the components in the solution are always equal to unity [S1]. To go from molar concentrations to equivalent fractions, we transform equation (S6) into the following form:

$$\frac{\left(\frac{|z_2||z_m|\bar{c}_2Q_m}{|z_m|Q_m}\right)^{1/|z_2|}}{\left(\frac{|z_1||z_m|\bar{c}_1Q_m}{|z_m|Q_m}\right)^{1/|z_1|}}=K_{21}\frac{\left(\frac{|z_2||z_3|c_2c_3}{|z_3|c_3}\right)^{1/|z_2|}}{\left(\frac{|z_1||z_3|c_1c_3}{|z_3|c_3}\right)^{1/|z_1|}}, \tag{S8}$$

where z_m and Q_m are the charge number and concentration of fixed groups of the membrane, respectively, z_3 and c_3 are the charge number and concentration of coions, respectively.

The equivalent fractions of counterions in the membrane and in the solution will be equal to $\frac{|z_i|\bar{c}_i}{|z_q|Q}=\bar{\theta}_i$ and $\frac{|z_i|c_i}{|z_A|c_A}=\theta_i$, respectively:

$$\frac{\left(\bar{\theta}_2|z_m|Q_m\right)^{1/|z_2|}}{\left(\bar{\theta}_1|z_m|Q_m\right)^{1/|z_1|}}=K_{21}\frac{\left(\theta_2|z_3|c_3\right)^{1/|z_2|}}{\left(\theta_1|z_3|c_3\right)^{1/|z_1|}}\rightarrow\frac{\bar{\theta}_2^{\,1/|z_2|}}{\bar{\theta}_1^{\,1/|z_1|}}=K_{21}\left(\frac{|z_m|Q_m}{|z_3|c_3}\right)^{1/|z_1|-1/|z_2|}\frac{\theta_2^{\,1/|z_2|}}{\theta_1^{\,1/|z_1|}}, \tag{S9}$$

where $K_{21}\left(\frac{|z_m|Q_m}{|z_3|c_3}\right)^{1/|z_1|-1/|z_2|}=\bar{K}_{21}$ is the ion exchange equilibrium coefficient [S1]. From this follows the final form of the relation, which describes the ion-exchange equilibrium between the phases of the membrane and the solution:

$$\frac{\bar{\theta}_2^{\,1/|z_2|}}{\bar{\theta}_1^{\,1/|z_1|}}=\bar{K}_{21}\frac{\theta_2^{\,1/|z_2|}}{\theta_1^{\,1/|z_1|}}. \tag{S10}$$

References

S1. Helfferich, F. *Ion Exchange*; McGraw-Hil.; London and New York, 1962;

S2. Scholz, F. Nikolsky's ion exchange theory versus Baucke's dissociation mechanism of the glass electrode. *J. Solid State Electrochem.* **2011**, *15*, 67–68, doi:10.1007/s10008-010-1163-0.
