# Supplementary figures and images for: Mathematical Modeling of Monovalent Permselectivity of a Bilayer Ion-Exchange Membrane as a Function of Current Density
Source: Int J Mol Sci. 2022 Apr 24;23(9):4711. doi: 10.3390/ijms23094711 (PMC9104382; doi:10.3390/ijms23094711)

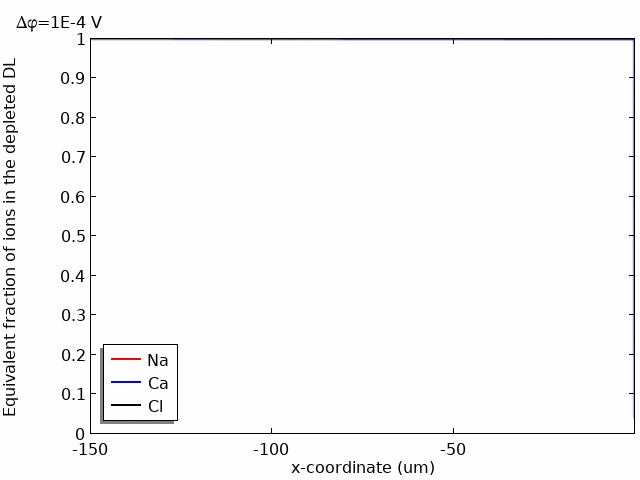

Supplement: Supplementary file 1 [file ijms-23-04711-s001.zip › Animation S1.gif]
